# Adagrasib: A landmark in the KRAS^G12C^‐mutated NSCLC

**DOI:** 10.1002/mco2.190

**Published:** 2022-11-25

**Authors:** He Tian, Zhenlin Yang, Jie He

**Affiliations:** ^1^ Department of Thoracic Surgery National Cancer Center/National Clinical Research Center for Cancer/Cancer Hospital Chinese Academy of Medical Sciences and Peking Union Medical College Beijing China

1

A recent study published in the *New England Journal of Medicine* by Pasi A. Jänne et al.^1^ presented the Phase II cohort results from a clinical trial (KRYSTAL‐1, NCT03785249), which evaluated adagrasib (MRTX849, a KRASG12C inhibitor) in KRASG12C‐mutated nonsmall‐cell lung cancer (NSCLC) previously treated with chemotherapy and antiprogrammed death 1 (PD‐1) or antiprogrammed death ligand 1 (PD‐L1) therapy. Adagrasib showed encouraging efficacy and acceptable safety, offering novel therapeutic avenues for KRASG12C‐mutated NSCLC (Figure 1A).

**FIGURE 1:**
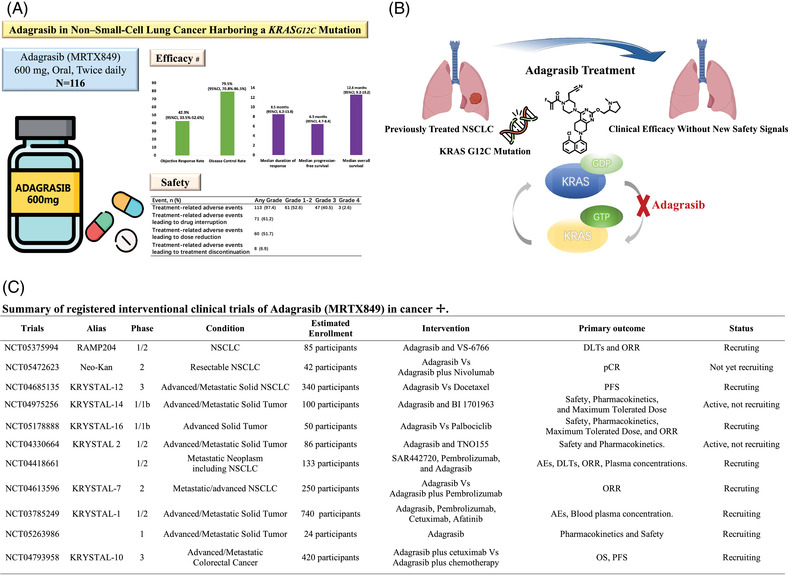
Adagrasib in KRASG12C‐mutated NSCLC. (A) A summary of the study by Pasi A. Jänne et al. #The median duration of response was based on the 48 patients with a response among all the 116 patients enrolled in the trial. The median progression‐free survival and the median overall survival were based on the 112 evaluable patients among all the 116 patients enrolled in the trial. This panel was generated with the help of Flaticon (https://www.flaticon.com/). (B) The administration of Adagrasib achieved clinical efficacy without new safety signals in patients with previously treated KRASG12C‐mutated NSCLC. NSCLC, nonsmall cell lung cancer. This panel was generated on Biorender.com. (C) A summary of registered interventional clinical trials of Adagrasib (MRTX849) in cancer. ✢The information in this summary was from ClinicalTrials.gov (https://clinicaltrials.gov/), which is a Web‐based resource providing information on publicly and privately supported clinical trials and is maintained by the National Library of Medicine (NLM) at the National Institutes of Health (NIH). NSCLC, nonsmall cell lung cancer. PFS, progression‐free survival. pCR, pathologic complete response. VS‐6766, a dual inhibitor of MEK and RAF. AEs, adverse events. ORR, objective response rate. BI 1701963, a pan‐KRAS inhibitor preventing the association of KRAS with son of sevenless homolog 1 (SOS1). DLTs, dose‐limiting toxicities. Palbociclib, a small molecule inhibitor of CDK 4 and 6. TNO155, a selective, orally bioavailable allosteric inhibitor of wild‐type SHP2.

This study enrolled 116 patients with histologically confirmed unresectable or metastatic NSCLC with KRASG12C mutation who had accepted treatment with at least one platinum‐based chemotherapy regimen and immune checkpoint inhibitors (ICIs) therapy. 98.3% of the patients accepted both chemotherapy and immunotherapy previously. The patients received a 600‐mg dose of adagrasib twice daily, with a fasted state. Among 112 patients with measurable disease at baseline, the confirmed objective response rate (ORR) was 42.9%. The median response duration of 48 patients with a response was 8.5 months (95% confidence interval [CI], 6.2 to 13.8), and the median progression‐free survival (PFS) among 112 evaluable patients was 6.5 months (95% CI, 4.7 to 8.4). The median overall survival (OS) was 12.6 months (95% CI, 9.2 to 19.2), with updated data on January 15, 2022. In 33 patients with radiographically evaluable CNS metastases, the intracranial confirmed ORR was 33.3% (95% CI, 18.0 to 51.8). 97.4% of the patients underwent treatment‐related adverse events, the most common of which were diarrhea, nausea, vomiting, and fatigue, leading to a 6.9% drug discontinuation rate. The ORRs were similar across PD‐L1 expression subgroups, indicating that the efficacy of adagrasib effectiveness was not affected by PD‐L1 expression. The ORRs in patients with coalterations in STK11, KEAP1, TP53, and CDKN2A were satisfying (range 28.6%‐58.3%). While the ORR in those who had STK11 wildtype with KEAP1 comutation was lower (14.3%). More evidence on this issue is warranted since precise stratification of KRAS‐mutated NSCLC patients is necessary before clinical decisions.

Adagrasib is a robust, orally available, small molecule covalent inhibitor binding to the KRASG12C cysteine 12 residue, locking the protein in its inactive GDP‐bound conformation, thus blocking KRAS‐dependent signaling and exerting antitumor efficacy in tumor models^2^ (Figure 1B). The registered interventional clinical trials of adagrasib in cancer were summarized (Figure 1C). As the first KRASG12C inhibitor approved by FDA (Food and Drug Administration), sotorasib yielded a durable clinical benefit in KRASG12C‐mutated NSCLC (NCT03600883). In NSCLC, adagrasib was superior to sotorasib in ORR (sotorasib 37.1%, adagrasib 42.9%), and they were similar in median OS (sotorasib 12.5 months, adagrasib 12.6 months) and median PFS (sotorasib 6.8 months, adagrasib 6.5 months) (NCT03785249 and NCT03600883). In addition, pharmacologic differences between adagrasib and sotorasib are the drug half‐life, dose‐dependent extended exposure with adagrasib, and potential central nervous system (CNS) penetration of adagrasib. Approximately 27%‐42% of KRASG12C‐mutated NSCLC patients have CNS metastases at diagnosis and are of dismal prognosis. Under the Response Assessment in Neuro‐Oncology Brain Metastases (RANO‐BM) criteria, Jänne et al.^1^ identified 42 patients with CNS metastases at baseline, the median intracranial PFS of whom was 5.4 months (95% CI, 3.3. to 11.6). A total of 33 patients of them could be evaluated radiographically, the intracranial confirmed ORR was 33.3% (95% CI, 18.0 to 51.8), and the median duration of intracranial response was 11.2 months (95% CI, 2.99 to not evaluable). In a previous report, sotorasib led to a 13% intracranial response rate in 16 KRASG12C‐NSCLC patients with stable, treated CNS metastases (CodeBreaK 100, NCT03600883). However, the CNS penetration ability of adagrasib warrants further investigation since all patients with CNS metastases recruited were previously treated and neurologically stable, and the number of patients with CNS metastases was limited. The good news is that at the 2022 American Society of Clinical Oncology (ASCO) Annual Meeting, Mirati Therapeutics Inc announced the first clinical data demonstrating CNS‐specific activity of KRASG12C inhibitor (adagrasib) in NSCLC patients with active and untreated CNS metastases (the Phase Ib cohort of NCT03785249): the intracranial ORR was approximately 32% under RANO‐BM criteria and the intracranial disease control rate was about 84% (NCT03785249, Meeting Abstract | 2022 ASCO Annual Meeting II). The adoption of patients with active CNS disease in Jänne et al.’s study also received attention from Kotecha et.al, who gave their concerns and suggestions about this issue.^3^ We expect more evidence derived from large‐scale cohorts. The efficacy comparisons we made between adagrasib and sotorasib were preliminary work based on the current data from different cohorts. More solid evidence about these comparisons should be referred to head‐to‐head RCTs (randomized control trials).

In NSCLC, the KRASG12C mutation frequency is 14% in adenocarcinoma and 0.5%–4% in squamous carcinoma.^1^ Although most patients in the study of Jänne et al.^1^ were ECOG 1 and had previously received both chemotherapy and ICI therapy, the efficacy of adagrasib was better than traditional second‐line chemotherapy. In NSCLC second‐line docetaxel treatment, the ORR was 14%, the PFS was 3.0 months (IQR 1.4‐6.9), and the median OS was 9.1 months (IQR 4.2‐18.0) (NCT01168973). Therefore, adagrasib could bring hope to a substantial late‐stage NSCLC patient irresponsive to traditional chemotherapy. We believe that the clinical outcome of adagrasib would be improved when applied at an earlier stage. The safety of adagrasib is acceptable. Although treatment‐related adverse events were observed in 97.4% of the patients, the drug discontinuation rate was low (6.9%). The most common events were gastrointestinal‐related events, similar to other KRASG12C inhibitors. We expect the ongoing evaluation of an additional dose level (400 mg orally twice daily)^1^ which might offer an opportunity to relieve the adverse events.

Moreover, KRASG12C mutation occurs in 3%‐4% of CRC (Colorectal Cancer). Durable blockade of KRASG12C might be of significance in CRC. In 2021, Weiss et al. reported that adagrasib demonstrated promising clinical efficacy and safety in KRASG12C ‐mutant CRC, suggesting the extensive use of this medicine (NCT03785249).

Neoadjuvant immunotherapy has improved the prognosis of NSCLC in the past five years, while resistance is an issue that cannot be bypassed. It is never too early to deliberate the combined utilization of KRASG12C inhibitors and immunotherapy. A meta‐analysis in 2022 reported that anti‐PD‐(L)1 therapy with or without chemotherapy achieved more prolonged survival than mono‐chemotherapy for KRAS‐mutant NSCLC (https://doi.org/10.1007/s00262‐021‐03031‐1). Therefore, combining ICIs and KRASG12C‐targeted medicine seems an obvious approach. A preclinical study reported that promoting an antitumor environment and lasting cures were found in mice models treated with sotorasib combined with the anti‐PD‐1 regimen, supporting the strategy of the combination of ICIs and KRAS inhibitor.^4^ However, a recent study showed that the successful combination of KRASG12C inhibition (MRTX1257) and ICIs was not universal in the NSCLC tumor models, the synergistic benefit was only seen in the most immunogenic tumor models, and most tumor refractory to ICIs was not resensitized by combination with KRASG12C inhibition.^5^ Therefore, the confronting questions are: (1) How to select the NSCLC patients who might benefit from the combinational therapy of KRASG12C inhibitors and ICIs? (2) What are the underlying mechanisms and solutions of resistance in the combination of KRASG12C inhibitors and ICIs? Therefore, more efforts are warranted to explore the interactions between KRASG12C inhibitors and cancer immunity. We look forward to the results of KRYSTAL‐7 (NCT04613596), a Phase II study evaluating the efficacy and safety of adagrasib monotherapy and in combination with pembrolizumab in cohorts of patients with advanced/metastatic KRASG12C‐mutated NSCLC.

## AUTHOR CONTRIBUTIONS

J.H. and Z.Y. are responsible for the study design. H.T. is responsible for the literature collection and manuscript drafting. H. T. and Z.Y. are responsible for revising the manuscript. J.H. and Z.Y. provide the final approval of the version to be published. All authors read and approved the final manuscript.

## CONFLICT OF INTEREST

The authors declare no competing interests.

## ETHICS STATEMENT

Not applicable.

## Data Availability

No original data were generated in this work.
